# Lack of Early Inflammation Signs of Acute Compartment Syndrome in an Immunodeficient Patient

**DOI:** 10.4274/tjh.2015.0255

**Published:** 2017-06-01

**Authors:** Burcu Belen, Özlem Çakıcı, Melikşah Uzakgider, Haldun Öniz, Meral Türker, Berna Atabay, Barış Malbora, Levent Karapınar

**Affiliations:** 1 Tepecik Training and Research Hospital, Clinic of Pediatric Hematology and Oncology, İzmir, Turkey; 2 Tepecik Training and Research Hospital, Clinic of Pediatrics, İzmir, Turkey; 3 Tepecik Training and Research Hospital, Clinic of Orthopedics, İzmir, Turkey; 4 Tepecik Training and Research Hospital, Clinic of Pediatric Oncology, İzmir, Turkey; 5 Tepecik Training and Research Hospital, Clinic of Pediatric Hematology, İzmir, Turkey

**Keywords:** Acute compartment syndrome, inflammation, immune deficiency

Acute compartment syndrome (ACS) is defined as the continuous elevation of interstitial tissue pressure within an osteofascial envelope to nonphysiological levels. It can be reversible if it is recognized early; however, it may progress to permanent disability. Therefore, early recognition and treatment is critical for optimal outcomes [1]. Pain, pallor, paresthesia, paralysis, and pulselessness (the ‘five Ps’) are reliable symptoms of ACS; however, the lack of them may be challenging in immunodeficient patients [2,3].

Here we present upper extremity ACS in an 18-year-old male patient with non-Hodgkin lymphoma. He was admitted with antecubital vein thrombosis during gram-negative sepsis without overt signs of inflammation in the affected arm while he was neutropenic. With the increase in white blood cells, first inflammatory findings of cellulitis and soon after that upper extremity ACS became evident (Figure 1). The ACS was assumed to be caused by the increased pressure of the compartment following superficial thrombosis that may have led to obstruction of venous flow accompanied by cellulitis of the forearm. Front forearm fasciotomy was performed with primary fixation of the ruptured flexor digitorum profundus muscle in combination with intravenous antibiotherapy (Figure 2). Due to lack of initial inflammation signs in immunodeficient patients, ACS diagnosis is particularly difficult. Early recognition and expeditious surgical treatment are essential to obtain a good clinical outcome and prevent permanent disability.

## Figures and Tables

**Figure 1:**
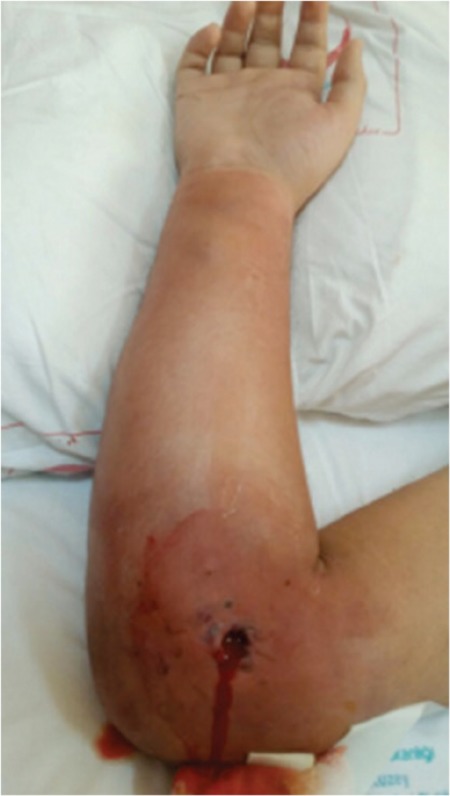
Acute compartment syndrome of upper extremity after restoration of white blood cells.

**Figure 2:**
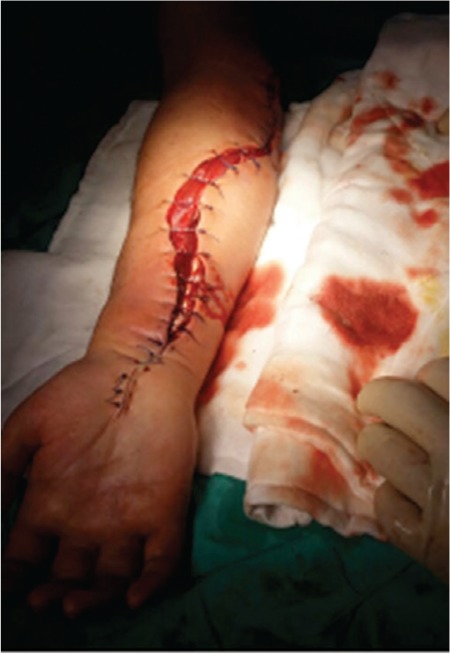
Front forearm fasciotomy was performed for treatment of acute compartment syndrome.
